# The Evaluation of the BioFire FilmArray Meningitis/Encephalitis Panel for the Detection of Bacteria and Yeast in Cerebrospinal Fluid Specimens

**DOI:** 10.7759/cureus.56260

**Published:** 2024-03-16

**Authors:** Yassine Ben Lahlou, Yassine Eddair, Yao Christian H Dokponou, Mostapha Elouennass, Mariama Chadli

**Affiliations:** 1 Bacteriology, Mohammed V Military Training Hospital, Mohammed V University, Rabat, MAR; 2 Neurosurgery, Mohammed V Military Training Hospital, Mohammed V University, Rabat, MAR

**Keywords:** neisseria meningitidis, cerebrospinal fluid (csf), polymerase chain reaction (pcr), meningitis, biofire filmarray meningitis/encephalitis panel

## Abstract

Background and objective

Infectious meningitis and encephalitis are serious diseases that can have fatal consequences, especially in the case of bacterial meningitis. Molecular biology has made it possible to quickly introduce appropriate treatment.

Our study aims to evaluate the FilmArray Meningitis/Encephalitis Polymerase Chain Reaction (PCR) Panel (BioFire Diagnostics, Salt Lake City, Utah) implemented in our department compared to traditional methods.

Material and methods

This was a retrospective single-center study conducted in the Department of Bacteriology of Mohammed V Military Training Hospital, Rabat, for a period of four years. All cerebrospinal fluid (CSF) samples from patients with symptoms of meningitis or meningoencephalitis submitted to the laboratory for cytobacteriological analysis were included in the study. Conventional analysis has been compared with molecular biology.

Results

The overall agreement rate with FilmArray in our study was 86%. The sensitivity to *Escherichia coli K1*, *Haemophilus influenzae*, *Neisseria meningitidis*, *Streptococcus agalactiae*, and *Streptococcus pneumoniae* was 100%. And for *Cryptococcus neoformans* it was 83% in our study.

Conclusion

In summary, this technique can be used to diagnose bacterial meningitis more sensitively than with conventional techniques, while at the same time allowing a rapid and efficacious patient's treatment.

## Introduction

Meningitis and meningoencephalitis are serious diseases of the central nervous system, sometimes carrying functional and vital prognoses. In addition to the impact on the patient, the cost of these infections is high, directly related to hospitalization, and indirectly to loss of contribution to society [1, 2]. In Morocco, bacterial meningitis remains a real public health problem [3]. It usually occurs in an endemic-sporadic pattern, with epidemic outbreaks of meningococcal and viral meningitis occurring [4].

Classically, diagnosis is based on staining techniques and cultures, which are time-consuming and sometimes lack sensitivity and specificity [5]. The FilmArray Meningitis/Encephalitis Polymerase Chain Reaction (PCR) Panel (ME PCR Panel; BioFire Diagnostics, Salt Lake City, Utah) is the first multiplex PCR technique that enables the simultaneous and rapid detection of 14 pathogens, including seven viruses and six bacteria. These include *Escherichia coli K1,* *Haemophilus influenzae*, *Listeria monocytogenes*, *Neisseria meningitidis*, *Streptococcus agalactiae*, *Streptococcus pneumoniae*, *cytomegalovirus* (CMV), e*nterovirus (EV), *herpes simplex virus 1 (HSV-1), herpes simplex virus​​​​​​​ 1​​​​​​2 (HSV-2), human herpesvirus 6 (HHV-6), human parechovirus (HPeV), varicella zoster virus​​​​​​​ (VZV), and *Cryptococcus neoformans*/*Cryptococcus gattii*.

Our study aims to evaluate this technique implemented in our department compared to traditional methods.

## Materials and methods

Study site

This was a retrospective single-center study conducted in the Department of Bacteriology, Mohammed V Military Training Hospital, Rabat. This is a university hospital with a capacity of 1000 beds and a center for infectious and tropical diseases.

Study period

The study lasted four years, from January 1st, 2020, to December 31st, 2023.

Inclusion criteria

All cerebrospinal fluid (CSF) samples from patients with symptoms of meningitis or meningoencephalitis submitted to the laboratory for cytobacteriological analysis were included in the study.

Bacteriological analysis

All included samples were subjected to conventional analysis (direct examination and culture). Cultures were performed on blood agar and PolyViteX chocolate agar, with spot inoculation and enrichment on heart/brain medium and subculture on blood medium the following day. *Cryptococcus neoformans *was detected with India ink staining and cultured on Sabouraud medium in addition to the other common media. Bacterial isolates were identified using standard biochemical and phenotypic techniques. Antibiograms were performed according to the recommendations of the French Society of Microbiology (SFM).

FilmArray molecular biology analysis

Molecular biology analysis was performed on samples whose cytology was suggestive of meningitis. A cytological threshold of 20 leukocytes/mm3 was established. In immunocompetent patients, the FilmArray testing procedure was performed according to the manufacturer's instructions.

Only bacteriological and *Cryptococcus neoformans* results were analyzed in this study. The results were compared with the patient's clinical data. The limit of the study was the impact of using this tool on the therapeutic management of the patient, especially in terms of time, and also the impact on mortality.

Statistical analysis

The SPSS 2023 version (IBM Inc., Armonk, New York) was used for statistical analysis.

## Results

A total of 208 CSF were tested by FilmArray during the four-year study period. Twenty-six (12.5%) were positive for bacteria or yeast. Considering FilmArray results, *Streptococcus pneumoniae* was the most frequently detected pathogen with 11 cases, followed by* Neisseria meningitidis* with four cases,* Streptococcus agalactiae* with three cases, two cases of *Haemophilus influenzae *and a single case of *Escherichia coli K1*. No cases of *Listeria monocytogenes* were identified. Only one case of meningitis with the combination of *Escherichia coli K1* and *Streptococcus pneumoniae* was identified. The average age of FilmArray-positive patients was 44 years. Table 1 shows the results of the FilmArray tests compared to conventional methods, as well as the results of cytology, CSF glucose, and CSF protein.

**Table 1:** Results of FilmArray testing compared with conventional methods WBC - white blood cells (/mm3); NR - not realized; *S. pneumoniae* - *Streptococcus pneumoniae; E. coli *- *Escherichia coli*;* C. neoformans *- *Cryptococcus neoformans*;* N. meningitidis - Neisseria meningitidis*;* H. influenzae - Haemophilus influenzae*;* S. agalactiae - Streptococcus agalactiae*; GNB - gram negative bacilli

Sample	WBC count	Culture	FilmArray	Soluble antigens	Gram staining	Glucorachia, g/l	Proteinorachia, g/l
1	1600	S. pneumoniae	S. pneumoniae	NR	Numerous gram-positive diploid cocci	0.37	2.79
2	16000	S. pneumoniae	S. pneumoniae	NR	Numerous gram-positive diploid cocci	0.01	7.85
3	235	S. pneumoniae	S. pneumoniae	NR	Numerous gram-positive diploid cocci	0.01	8.78
4	2602	S. pneumoniae	S. pneumoniae	NR	Numerous gram-positive diploid cocci	0.01	4.87
5	197	S. pneumoniae	S. pneumoniae	NR	Numerous Gram-positive diploid cocci	0.01	6.39
6	1350	S. pneumoniae	S. pneumoniae	Negative	No bacterial flora	0.62	2.57
7	14400	S. pneumoniae	S. pneumoniae	NR	Numerous gram-positive diploid cocci	0.01	11.25
8	132	S. pneumoniae	S. pneumoniae	NR	Numerous gram-positive diploid cocci	0.01	8.39
9	1260	S. pneumoniae	S. pneumoniae	NR	Numerous gram-positive diploid cocci	0.01	5.17
10	3790	S. pneumoniae	S. pneumoniae	S. pneumoniae	Numerous gram-positive diploid cocci	0.39	2.73
11	197	S. pneumoniae + E. coli	S. pneumoniae + E. coli	NR	Numerous gram-positive diploid cocci + GNB	0.01	6.39
12	6	C. neoformans	C. neoformans	NR	Many yeasts	0.13	0.67
13	144	C. neoformans	Negative	NR	Many yeasts	0.31	4.27
14	19	C. neoformans	C. neoformans	NR	No bacterial flora/ no yeast	0.23	1.01
15	2	C. neoformans	C. neoformans	NR	Many yeasts	0.47	0.27
16	12	C. neoformans	C. neoformans	NR	Many yeasts	0.74	0.22
17	120	C. neoformans	C. neoformans	NR	Many yeasts	0.23	5.54
18	970	N. meningitidis	N. meningitidis	NR	Numerous gram-negative diploid cocci	0.59	2.63
19	278	Sterile	N. meningitidis	NR	Numerous gram-negative diploid cocci	0.63	1.07
20	13720	Sterile	N. meningitidis	*N. meningitidis* ACY/W135	Rare gram-negative diploid cocci	0.91	6.72
21	52	Sterile	N. meningitidis	*N. meningitidis* ACY/W135	Numerous gram-negative diploid cocci	0.81	0.89
22	4373	H. influenzae	H. influenzae	NR	Numerous gram-negative bacilli	0.46	7.74
23	580	Sterile	H. influenzae	NR	No bacterial flora	0.23	0.49
24	50	Sterile	S. agalactiae	NR	No bacterial flora	0.18	12.82
25	4300	Sterile	S. agalactiae	NR	Numerous gram-positive diploid cocci	0.07	9.79
26	610	S. agalactiae	S. agalactiae	S. agalactiae	Rare gram-positive diploid cocci	0.63	2.67

The overall agreement rate with FilmArray in our study was 86%. The sensitivity to *Escherichia coli K1*, *Haemophilus influenzae*, *Neisseria meningitidis*, *Streptococcus agalactiae*, and *Streptococcus pneumoniae* was 100%. And for *Cryptococcus neoformans *it was 83% in our study (Table 2).

**Table 2:** Germ sensitivity and specificity *S. pneumoniae - Streptococcus pneumoniae*; *N. meningitidis - Neisseria meningitidis*; *S. agalactiae - Streptococcus agalactiae*; *H. influenzae - Haemophilus influenzae*; *E. coli - Escherichia coli*; *C. neoformans - Cryptococcus neoformans*

Culture	Sensitivity	Specificity
S. pneumoniae	100%	100%
N. meningitidis	100%	97%
S. agalactiae	100%	98%
H. influenzae	100%	99%
E. coli	100%	100%
C. neoformans	83%	100%

## Discussion

During the study period, a total of 208 CSF were tested by FilmArray, with a bacterial positivity rate of 12.5%. This rate is comparable to that reported in the literature [6]. In our series, *Streptococcus pneumoniae* and *Neisseria meningitidis* accounted for 42.5% and 15.5%, respectively. These are the two species most commonly responsible for bacterial meningitis worldwide, involved in 25.1-41.2% and 9.1-36.2% of cases, respectively [7]. In our study, *Streptococcus agalactiae* accounted for 11.3%. This is a common cause of meningitis in newborns, especially those born prematurely. Of our three patients detected by FilmArray, only one was a newborn. Two cases of *Haemophilus influenzae* were identified. It accounts for 0.2 to 15.5% of bacterial meningitis cases worldwide [8]. Since the introduction of the vaccine in 2007, it has become a rare cause of meningitis [4].

Bacterial meningitis, especially purulent meningitis, represents a diagnostic, therapeutic, and biological emergency because it can jeopardize the vital and functional prognosis. Classically, diagnosis is based on culture, and we used this method as a reference for comparison. The overall concordance rate with FilmArray in our study was 86%, while a similar study reported a concordance rate of 88% [9]. For *Escherichia coli K1*, *Haemophilus influenzae*, *Neisseria meningitidis*, *Streptococcus agalactiae*, and *Streptococcus pneumoniae*, the sensitivity was 100%. For* Cryptococcus neoformans* it was 83% in our study. According to the literature, all pre- and post-marketing studies found high and specific specificity [10-12].

Specifically, 11 cases of *Streptococcus pneumonia* and a single case of *Escherichia coli K1* were detected by both culture and FilmArray. *Streptococcus agalactiae* was detected by FilmArray in three cases, while culture diagnosis was possible in only one case. This indicates either a true positive or a false positive. In these three patients, cytology was conclusive, and the clinical record concluded that it was meningitis, which suggests a real positive and, therefore, high sensitivity of the FilmArray compared to culture. However, A prospective, multicenter, FilmArray-based panel evaluation study of 1560 CSF revealed seven false-positive results for *Streptococcus pneumoniae* and one false-positive result for *Streptococcus agalactiae* [5].

*Neisseria meningitidis* was detected by the FilmArray technique in four patients, only one by culture, but all with significant cytology and a predominance of neutrophils and clinical data suggestive of meningitis. Two cases of *Haemophilus influenzae* were detected by FilmArray, whereas only one case was diagnosed by culture. This confirms the high sensitivity of this technique compared to culture, even if the latter is still the reference technique [5]. Its sensitivity depends on the type of pauci bacterial sample, the antibiotic used, and the pathogen. *Neisseria meningitidis* and *Haemophilus influenzae* are two sensitive germs that absolutely require supplementation of media with factors for growth [13].

In our study, FilmArray was used to detect *Cryptococcus neoformans *in six cases, while culture was positive in seven cases. All of these results concerned HIV patients in the AIDS stage. Our false negative was a patient with a positive India ink staining and culture. This false negative problem was already highlighted in the test's pre-marketing study, in which seven of the eight samples that were positive using the immunochromatographic technique (antigen detection) were using the BioFire® ME test, and another PCR comparator was found to be negative [14]. Other studies also confirm this problem [15, 16]. Molecular biology is not suitable for diagnosing cryptococcal meningitis, for which the reference method is antigen testing [14, 17]. This false-positive problem could be explained by antifungal treatment, the low concentration of the pathogen, and the high detection threshold of the FilmArray technique [18].

No cases of *Listeria monocytogenes* have been detected. However, this germ is of particular interest because it is naturally resistant to third-generation cephalosporins, which is the recommended treatment in these cases of meningitis. Studies have shown high sensitivity for FilmArray detection [19,20].

Compared to gram staining, the technique also has a high sensitivity, as direct examination after staining only enabled a diagnosis in 22 cases (84%). The sensitivity of gram stain correlates with the concentration and type of bacteria, as well as the speed at which the sample is delivered to the laboratory and the experience of the investigator [21]. This also applies to the soluble antigen test, which was only used to make the diagnosis in four cases (15.5%).

Several studies confirm that the FilmArray technique has high sensitivity compared to culture [5, 6, 9, 16, 17], a fact confirmed in our study. This technique is not influenced by the antibiotic taken [22, 23] and does not require viable bacteria, making it easy to perform and, in addition, allowing the detection of a wide range of bacteria, viruses, and yeasts. Its rapidity provides results in less than two hours and, therefore, has a direct influence on therapeutic decision-making. In addition to the patient, the focus is on the impact on the community since, in the event of meningitis caused by *Neisseria meningitidis*, prophylaxis for the surrounding community can be carried out as quickly as possible.

However, the limitation of this technique remains to be the inability to detect pathogens not included in the panel, particularly those involved in nosocomial meningitis. Another limitation is cost, especially in a developing country. Although the direct cost of the analysis is higher than that of routine methods, several studies have confirmed that this technique can be used to detect a wide range of pathogens, allows for shorter hospital stays, allows for more frequent use of narrow-spectrum targeted antibiotic therapy, and reduces the number of prophylactic treatments initiated, resulting in significant potential savings per patient [24-27].

Despite these strengths, in our opinion, this technology can never replace conventional methods. An analysis of bacteriological and biochemical data, combined with a comparison with clinical elements, allows a final decision to be made. Overall, each institution can set its own algorithm to define the place of this panel in the diagnostic strategy.

## Conclusions

The FilmArray technique is a fast, easy-to-use technique that enables targeted research around the clock but can never replace traditional methods. The dialogue between clinician and biologist remains the cornerstone of patient management and, above all, of assessing the value of the technology with its cost. Further prospective studies are needed to define the place of this technique in the diagnostic strategy.
